# Perceived cognitive performance in off‐prescription users of modafinil and methylphenidate: an online survey

**DOI:** 10.1002/brb3.3403

**Published:** 2024-02-04

**Authors:** Rachel D. Teodorini, Nicola Rycroft, James H. Smith‐Spark

**Affiliations:** ^1^ Division of Psychology, School of Applied Sciences London South Bank University London UK

**Keywords:** cognitive performance, methylphenidate, modafinil, off‐prescription

## Abstract

**Introduction:**

Modafinil and methylphenidate are used off‐prescription for cognitive enhancement in healthy individuals. Such use is often reported in online surveys but it is unclear whether drug use for cognitive enhancement is motivated by perceived poor cognitive performance or a desire to improve good cognitive performance. The current study investigated whether off‐prescription users of modafinil and methylphenidate differed in their self‐perceived cognitive performance from people who do not take these drugs.

**Method:**

An online survey targeting forum sites assessed self‐perceived cognitive function via the Adult Attention Deficit/Hyperactivity Disorder Self‐Report Scale, the Cognitive Failures Questionnaire, and the General Procrastination Scale.

**Results:**

There were 249 respondents, of whom 43% reported no use of modafinil and methylphenidate (the control group) and 58% reported use of one or both drugs without a prescription for cognitive enhancement. This created an independent samples design with three groups. On both the Adult Attention Deficit/ Hyperactivity Disorder Self‐Report Scale and General Procrastination Scale, modafinil and methylphenidate users reported higher scores than the control group, indicating higher levels of perceived inattention and procrastination. Scores on the Cognitive Failures Questionnaire indicated that modafinil and methylphenidate users rated themselves as having fewer cognitive failures than controls.

**Conclusion:**

These findings suggest that at least some reported off‐prescription users of modafinil and methylphenidate may be seeking to reduce the impact of self‐perceived poorer performance, particularly in forms of cognition that are likely to impact on self‐directed or self‐motivated work.

## Abstract 2

The current study investigated whether off‐prescription users of modafinil and methylphenidate differed in their self‐perceived cognitive performance from people who do not take these drugs. An online survey targeting forum sites assessed self‐perceived cognitive function via the Adult Attention Deficit/Hyperactivity Disorder Self‐Report Scale, the Cognitive Failures Questionnaire, and the General Procrastination Scale. Modafinil and methylphenidate users reported higher scores than the control group, indicating higher levels of perceived inattention and procrastination suggesting that these modafinil and methylphenidate users may be self‐medicating for self‐perceived inattention and procrastination.

## 1 INTRODUCTION

Cognitive enhancing drugs (CEDs) are prescribed for conditions such as attention deficit hyperactivity disorder (ADHD) and dementia (Lanni et al., 2008; Outhoff, 2016). The effectiveness of CEDs such as methylphenidate in treating ADHD is well‐recognized (Castells et al., 2011; Van der Oord et al., 2008). Benefits of CEDs in dementia have also been noted in patients with mild to severe dementia (Rattinger et al., 2013). However, the use of such drugs by healthy individuals for enhancing nonclinically impaired functions is growing internationally (Dursun et al., 2019; McCabe et al., 2005; Teodorini et al., 2020). Of the many CEDs available on the market, the two most discussed and noted for their positive effects on cognition in healthy adults are methylphenidate and modafinil (for reviews, see Dubljević & Ryan, 2015; Repantis et al., 2010). Motivations for using CEDs include to improve concentration, to reduce fatigue and to “get more done” (DeSantis et al., 2008; Faraone et al., 2020; Rabiner et al., 2009; Teodorini et al., 2020). However, less is known about whether CED use is motivated purely by enhancement of already good performance or to improve poorer cognitive functions. Answers to this question could help inform both the ethical debate about fairness and access to CEDs (Sahakian & Morein‐Zamir, 2011) and to help direct CED users to support for problems with attentional control. Therefore, the current online self‐report study investigated whether off‐prescription users of modafinil and methylphenidate may be self‐medicating for perceived poor cognitive functions.

The off‐prescription use of modafinil and methylphenidate has been reported in schools, universities and in the workplace (Aikins, 2011; Leon et al., 2019; Singh et al., 2014; Vargo & Petróczi, 2016). Modafinil and methylphenidate have different pharmacological profiles, clinical uses, and potentials for abuse (Ballon & Feifel, 2006; Jasinski, 2000; Minzenberg & Carter, 2008; Wood et al., 2014). Despite this, there has been a tendency to treat off‐prescription CED users as a homogenous group, even though they are using a wide variety of substances (Rubin‐Kahana et al., 2020), and moreover, reports of the benefits obtained from CEDs differ between users of modafinil and methylphenidate (Teodorini et al., 2020). A further aim of the current study was, therefore, to explore whether users of modafinil and methylphenidate differ from both each other and non‐CED users in their self‐perceived cognitive performance.

Undiagnosed problems with attention have been identified in community samples, with high levels of ADHD symptoms reported by 10% of college students without a formal diagnosis (Garnier‐Dykstra et al., 2010) but only a few studies to date have explored whether this figure is higher among people who use CEDs off‐prescription (Arria et al., 2011; Garnier‐Dykstra et al., 2010; Ilieva & Farah, 2019; Peterkin et al., 2011; Poulin, 2007). Poulin (2007) asked adolescents to complete the Ontario Child Health Study Hyperactivity Scale and found that 20.5% of this group tested positively for ADHD symptomatology, twice the figure reported by Garnier‐Dykstra et al. (2010). Furthermore, Poulin's (2007) respondents with a positive ADHD screening test had a 2.3‐fold increased likelihood of nonmedical use of methylphenidate, compared with those who tested negative on the ADHD screening test. Benson et al. (2018) used the Current Symptoms Scale to measure ADHD symptomology and reported that participants who met the criteria for ADHD were 2.90 times more likely to misuse stimulants than those who did not.

Other studies (e.g., Arria et al., 2011; Peterkin et al., 2011) used the World Health Organization (WHO) Adult ADHD Self‐Report Scale (ASRS) (Kessler et al., 2005) to assess ADHD symptoms. The Adult ASRS is a standardized and well‐validated tool for assessing adult ADHD symptoms (Gray et al., 2014). Peterkin et al. (2011) used the ASRS Part A to compare the nonprescribed use of ADHD medications for academic purposes among university students who did or did not screen positive for ADHD. Of the 39 respondents who reported misuse of ADHD medications for academic purposes, they found that 77% tested positive for ADHD symptoms, compared with just 10% of those who reported no misuse of ADHD medications. This high percentage is at odds with the 20.5% reported by Poulin (2007). These different percentages may reflect a greater level of undiagnosed ADHD in North Virginia, although the number of participants differed greatly between the Poulin's (2007) and Peterkin et al.’s (2011) studies. Again, using the ASRS, Arria et al. (2011) found that 17% students who reported regular off‐prescription use of stimulants were at high risk of ADHD compared to just 8% of nonusers. Similar results have also been reported by Rabiner et al. (2009) who found that nonmedical use of stimulants was associated with symptoms of inattention rather than hyperactivity. Ilieva and Farah (2019) also reported that the off‐prescription use of ADHD medication, including methylphenidate, related positively to self‐perceived attention problems measured by the Barkley and Murphy ADHD Symptom Checklist (Murphy & Barkley, 1995).

These links between CED use and self‐ perceived symptoms of ADHD arises from research that has mostly been conducted on student populations. To address this concern, therefore, the current study sought to extend this work through the recruitment of a more diverse sample via an online survey aimed at forum users across the world. In addition to investigating the link between CED use and ADHD symptomology, the current study also explored potential differences between CED users and nonusers in procrastination and everyday, real‐world, cognitive lapses. Given that previous work in this area has already suggested ADHD symptomology may be higher among student CED users (Arria et al., 2011; Ilieva & Farah, 2019; Peterkin et al., 2011; Rabiner et al., 2009), exploring whether these differences extend to other areas of real‐world cognitive control would help inform the understanding of why CEDs are used.

The frequency with which an individual makes absentminded errors has been found to vary due to individual differences and includes perceptual, action, and memory failures (Broadbent et al., 1982; Unsworth et al., 2012). Everyday cognitive slips are experienced by everyone, but these slips occur more frequently in individuals with conditions affecting cognition, such as dyslexia (Smith‐Spark et al., 2004), ADHD (Kim et al., 2014), and Parkinson's disease (Poliakoff & Smith‐Spark, 2008). These three studies used the 25‐item Cognitive Failures Questionnaire (CFQ) (Broadbent et al., 1982) to identify the frequency of cognitive failures within the past 6 months and the CFQ has good external validity (e.g., de Paula et al., 2017; Ekici et al., 2016; Kim et al., 2014; Poliakoff & Smith‐Spark, 2008; Smith‐Spark et al., 2004; Wallace & Vodanovich, 2003). If off‐prescription users of modafinil and methylphenidate are self‐medicating for poor cognitive performance, the CFQ may capture how such failures impact on everyday life.

In addition to inattention (as measured via self‐reports of ADHD symptoms) and cognitive failures, a common barrier to accomplishing tasks is procrastinatory behavior, defined by Steel (2007) as a conscious delay of a planned course of action, even though this delay is likely to have negative outcomes. Procrastination is noted as a problem particularly for both students (Rabin et al., 2011) and workers (Nguyen et al., 2013). As common motivations for using CEDs include to “avoid procrastination” and “to get more done” (Teodorini et al., 2020), “increased academic performance” (Fond et al., 2016) and “productivity” (Novak et al., 2007; Sharif et al., 2021), it remains an open question as to whether CED users report higher levels of procrastination than a comparable group of non‐CED users. The 20‐item General Procrastination Scale (GPS) (Lay, 1986) was developed to assess trait procrastinatory behavior. Using this scale, Ferrari and Sanders (2006) compared the self‐perceived levels of procrastination in patients with ADHD and healthy controls and found significantly higher rates of procrastination in the ADHD group. Niermann and Scheres (2014) recruited university students who had tested positive on a self‐report scale for ADHD and reported that ADHD‐related symptoms of inattention, but not hyperactivity or impulsivity, were positively correlated with procrastination. Procrastination has, therefore, not only been associated with ADHD but overcoming procrastination has also been noted as a motivation for using CEDs (Aikins, 2011; Teodorini et al., 2020).

Three questionnaires, the ASRS (Kessler et al., 2005), the CFQ (Broadbent et al., 1982), and the GPS (Lay, 1986) were, therefore, used in the current study to investigate whether modafinil and methylphenidate users may perceive themselves as experiencing cognitive problems or failures and may, therefore, be using modafinil and methylphenidate to alleviate these. Since higher rates of recreational drug use have been reported among CED users (McCabe et al., 2006) and some recreational drugs can impact upon cognitive performance (e.g., Indlekofer et al., 2009), questions were also asked about rates of use of the three most used recreational drugs, namely nicotine, alcohol, and cannabis (Hultgren et al., 2021). It was hypothesized that, compared with a non‐CED‐using group, CED users would self‐report worse performance on the ASRS and, given the link between ADHD and procrastination, would self‐report worse performance on the GPS. Additionally, as proposed earlier, if CED users were to be self‐medicating for poor cognitive performance, it is likely that this would be reflected in their everyday life; therefore, it was also hypothesized that CED users would self‐report worse performance on the CFQ compared with non‐CED users.

## 2 METHODS

### 2.1 Respondents

A convenience sample of CED users and non‐CED‐using controls were recruited via advertisements on two online forums, Bluelight (https://www.bluelight.org) and Reddit (https://www.reddit.com). For details of the sub‐Reddits used, see Supplementary File S1. No reward was offered for their participation. The survey was conducted between February 11, 2018 and November 17, 2018. The respondents who contributed data to the study had reported taking either modafinil and/or methylphenidate or reported never having taken either modafinil or methylphenidate (control group). The exclusion criteria consisted of being under the age of 18 and/or currently being under the influence of a psychoactive drug.

### 2.2 Materials

Qualtrics^XM^ survey software was used to design and administer the survey. In total, it contained 99 questions (although, depending on their responses, the participants were not required to answer every question). The estimated response time varied from 8 to 30 min. The survey was divided into a number of sections (detailed below).

### 2.3 Demographics

This section covered age, gender, nationality, and education details, whether respondents were currently engaged in study including vocational, continuing professional development and “high school/ A Level” in addition to university degrees.

### 2.4 Cannabis, nicotine, and alcohol use

This section comprised three subsections relating to cannabis, nicotine, and alcohol use, respectively. The questions in this section focused on current, frequent use of cannabis and nicotine. For the purpose of analyzing the data collected on cannabis use, a clearer understanding of the frequency of use of cannabis was required. Therefore, responses to the question “in the past 6 months how regularly have you taken cannabis” were condensed: “everyday/almost everyday,” “three to four times per week,” and “once per week” were grouped into the variable “once or more per week.” The responses “once or twice per month” and “up to three times in total” were grouped into the variable “less than once per week” and the response “none” was renamed “none in the past 6 months” for clarity.

In Section 3.2.4, the Alcohol Use Disorders Identification Test (AUDIT; Babor et al., 1992) was used to identify problematic alcohol use. The AUDIT is a 10‐item questionnaire created by the World Health Organization (WHO) as a brief screening tool to identify individuals with hazardous and harmful alcohol use behavior (Babor et al., 2001). Questions are presented with a 5‐point Likert scale response and total scores range from 0 to 40. Scores between 8 and 15 represent a medium level of self‐perceived alcohol problems and scores above 16 represent a high level of alcohol problems (Babor et al., 2001).

### 2.5 Modafinil and methylphenidate use

These two sections were devoted to modafinil and methylphenidate use. These sections asked about age of first use, doses used, and the usual route of administration of both drugs.

### 2.6 Adult ADHD Self‐Report Scale (ASRS‐V1.1)

The ASRS (Kessler et al., 2005) uses a 5‐point Likert scale response to indicate the frequency of occurrence of symptoms within the past 6 months, with scores on each item ranging from 0 to 4 (never = 0, rarely = 1, sometimes = 2, often = 3, and very often = 4). The ASRS is comprised 18 items; the first six items of the scale (Part A) are designed to screen adults for ADHD and consist of three questions addressing inattention and three questions addressing hyperactivity and impulsivity. Frequency scores for item 7 onward (Part B) are intended to provide additional information rather than serving as a diagnostic tool. Items 1–4 and 7–11 addressed inattention and all other items addressed hyperactivity and impulsivity. In the current study, the items were presented without any indication of what the questionnaire was measuring, so respondents were unaware that they were completing an ADHD questionnaire. The ASRS has been reported to have good test–retest reliability, with Cronbach's alpha of .885, followed by a 2‐week test–retest reliability Cronbach's alpha of .878 (Kim et al., 2013). The Cronbach's alpha for the current study was .85.

### 2.7 Cognitive Failures Questionnaire (CFQ)

The 25‐item CFQ (Broadbent et al., 1982) was presented with a 5‐point Likert scale response with each response scored as “very often” = 4, “quite often” = 3, “occasionally” = 2, “very rarely” = 1, and “never” = 0. Total scores range from 25 to 100, with higher scores indicating higher levels of susceptibility to cognitive slips. The questionnaire has good test–retest reliability. Broadbent et al.’s (1982) study reported two groups: one retesting after 21 weeks gave a correlation of *r* = .824 (*n* = 57) and the other one retesting after 65 weeks gave a correlation of *r* = .803 (*n* = 32). Additionally, Broadbent et al. (1982) reported the results of a sample of 98 women between the ages of 20 and 40 years, with the coefficient alpha in this case being .89, demonstrating good internal consistency. The Cronbach's alpha for the current study was .85.

Broadbent et al. (1982) argued that the CFQ provides a measure of general cognitive failure, which is important for external validity, as the individual's view of themselves is also shared by those who know them. While Broadbent et al. (1982) did consider whether a total score or individual item score would be more appropriate by performing a factor analysis, the factors found could not be replicated and it was concluded that the CFQ's structure is unidimensional; thus using a total score of all items as representative. Later studies have also attempted to identify factors within the CFQ (e.g., Larson et al., 1997; Pollina et al., 1992; Wallace et al., 2002) but there has been no commonality between the studies in the factors so identified. That said, the only study that was retested and confirmed by confirmatory factor analysis (Wallace, 2004) was the analysis by Wallace et al. (2002), which found four factors, memory relating to memory errors and forgetfulness, distractibility relating to disruption of internally focused attention, blunders of a social nature, and names. Wallace's (2004) factors were used in the current study to resolve an issue with missing data as explained in Section 2.9.

### 2.8 General Procrastination Scale (GPS)

The 20‐item GPS (Lay, 1986) was also presented. Again, a 5‐point Likert scale was used, with each response being scored as “extremely untrue” = 1, “moderately untrue” = 2, “neutral” = 3, “moderately true” = 4, and “extremely true” = 5, although 10 of the items are reverse scored. Higher scores indicate higher levels of self‐perceived procrastination. Total scores range from 20 to 100 with higher scores indicating higher levels of procrastination. The GPS is a unidimensional questionnaire, and this was confirmed by Sirois et al. (2019), with a coefficient alpha of .82 (Lay, 1986) and Ferrari (1989) reported good test–retest reliability of .80. The Cronbach's alpha for the current study was .88.

### 2.9 Analysis

An independent‐sample design with three groups was used, a control group of respondents who reported never taking modafinil or methylphenidate, a “modafinil‐only” group who reported taking modafinil but not methylphenidate, and a methylphenidate group who reported taking methylphenidate and may or may not have taken other CEDs (including modafinil). Henceforth, these groups are referred to by their names (modafinil‐only, methylphenidate, and control). These groups were formed post hoc, based on the self‐reports provided by respondents.

Due to an error in Qualtrics, item 1 of all three questionnaires was not answered by some participants. For the ASRS, the data from 22 participants who did not provide a response to the first item (all from the control group) were removed from the analysis because the analysis requires all responses to all items. For the CFQ, the four‐factor model proposed by Wallace et al. (2002) was used and the mean score for all other items of the Distractibility factor was used to replace the missing score for 106 participants (all from the control group). A factor analysis was then performed to assess the robustness of this approach. For details of the factor analysis and scree plot, see Supplementary File S2.

For the GPS, item 1 was removed for all participants to ensure equivalency between groups and the scores reported below are based on the remaining 19 questions. Different strategies were used to resolve this Qualtrics error because different scoring methods for the questionnaires and different questionnaire factor structures required different approaches to be taken for each questionnaire. Data relating to performance on the ASRS, CFQ, and GPS were analyzed using SPSS software, version 21. Kruskal–Wallis tests (and post hoc Mann–Whitney *U* tests) were performed on the complete ASRS, the ASRS inattentive scores and the hyperactive/impulsive scores under three conditions of group type (detailed above), the CFQ and GPS scores under three conditions of group type.

Kruskal–Wallis and post hoc Mann–Whitney *U* tests were also performed on self‐perceived nicotine and alcohol use under three conditions of group type. A log‐linear test was conducted on educational status and chi‐square analyses were conducted on cannabis use under the three conditions of group type.

### 2.10 Procedure

Ethical approval was granted for this study by the School of Applied Sciences Research Ethics Committee at London South Bank University (SAS1733). With permission from the forum moderators, an advertisement and link to the survey were posted on the selected forum sites. The advertisement detailed the nature of the survey and invited individuals to participate if they had taken either modafinil or methylphenidate and also if they had not taken either drug. Following a brief and informed consent, questions relating to demographic information were presented first, followed by questions asking about modafinil and methylphenidate use and then the ASRS, CFQ, and GPS.

## 3 RESULTS

### 3.1 Participant characteristics

The final sample size was 249, following the removal of the data from 76 respondents who reported having been prescribed either modafinil or methylphenidate.

A total of 35% (*N* = 86) of respondents reported that they took modafinil‐only and 23% (*N* = 57) indicated that they took methylphenidate and may or may not have taken other CEDs (only 8%, *N* = 19, reported taking methylphenidate‐only). The remaining 43% (*N* = 106) of respondents reported that they had not taken either modafinil or methylphenidate.

### 3.2 Demographic information

#### 3.2.1 Gender, age, and nationality

The majority of both the modafinil‐only group (79%, *N* = 68) and the methylphenidate group (77%, *N* = 44) identified as being male, whereas the control group reported roughly equal numbers of males (55%, *N* = 58) and females (43%, *N* = 46). The mean age for the modafinil‐only group was 29 years (SD = 9.91, range = 50, minimum = 18, maximum = 68), compared with the methylphenidate group, which was 25 years (SD = 6.79, range = 25, minimum = 18, maximum = 43) and the control group, which was 27 years (SD = 8.61, range = 37, minimum = 18, maximum = 55).

There was a roughly equal number of modafinil‐only respondents who reported being North American (28%, *N* = 24) or British (27%, *N* = 23), whereas a greater number of the methylphenidate group reported being North Americans (35%, *N* = 20) compared with those who reported being British (16%, *N* = 9). There was also a greater number of the control group who reported being North American (36%, *N* = 38) compared with those who reported being British (13%, *N* = 14). See Table 1 for details of all reported nationalities.

**TABLE 1:** Nationalities by group.

Nationality	Modafinil‐only *N* (%)^a^	Methylphenidate *N* (%)^a^	Control *N* (%)^a^	
North American	24 (27.91)	20 (35.09)	38 (35.85)	
British	23 (26.75)	9 (15.79)	14 (13.21)	
Australian	12 (13.95)	3 (5.26)	9 (8.49)	
Canadian	2 (2.33)	4 (7.02)	7 (6.60)	
German	–	8 (14.04)	7 (6.60)	
Rest of Europe	11 (12.79)	8 (14.04)	18 (16.98)	
Rest of world		11 (12.79)	4 (7.02)	9 (8.49)

^a^ Percentages relate to group and not to the whole sample.

#### 3.2.2 Current educational status

In the modafinil‐only group, 42% (*N* = 36) reported that they were studying for a university degree. A similar pattern was found with the methylphenidate group: 39% (*N* = 22) reported that they were studying for a university degree. The control group showed a slightly lower number, with 20% (*N* = 21) reported that they were studying for a degree.

A log‐linear test was conducted for group type (modafinil‐only, methylphenidate, and control groups) and currently studying for a qualification, and the analysis produced a final model with a likelihood ratio of χ^2^ = 0.00, *p* = n.s., indicating that the model fitted the data well. The model indicated that there was no significant two‐way interaction between group type and current university study status, χ^2^(2) = 4.86, *p* = .088.

Full details of respondents reporting their current studies can be found in Table 2.

**TABLE 2:** Currently studying for a qualification.

	Modafinil‐only # *N* (%)^a^	Methylphenidate # *N* (%)^a^	Control # *N* (%)^a^	
Currently studying for a qualification				
Yes		41 (47.67)	33 (57.90)	27 (25.47)
No	45 (52.33)	24 (42.11)	79 (74.53)	
Course type				
Vocational	2 (2.33)	2 (3.51)	–	
CPD	3 (3.49)	3 (5.26)	–	
A Levels	–	6 (10.53)	6 (5.66)	
University bachelor's program	18 (20.93)	14 (24.56)	18 (16.98)	
University master's program	11 (12.79)	5 (8.77)	3 (2.83)	
Doctoral studies	7 (8.14)	3 (5.26)	–	

^a^ Percentages relate to group and not to the whole sample.

#### 3.2.3 Cannabis and nicotine

Please see Supplementary File S3 for cannabis and nicotine findings.

#### 3.2.4 Alcohol

Total scores on the AUDIT were highest in the control group, with a mean of 13.96 (SD = 4.71), followed by the methylphenidate group (mean = 7.30, SD = 5.08) and the modafinil group (mean = 6.40, SD = 4.44). Due to uneven group sizes, a Kruskal–Wallis test was performed on the total AUDIT scores to test for the effect of group type. The difference between the mean ranks of 92.29 (methylphenidate), 81.15 (modafinil‐only), and 178.17 (control) were significant, *H*(_2_) = 101.90, *p* < .001.

Post hoc Mann–Whitney *U* tests revealed that both the methylphenidate scores, *U* = 947.50, *N*_methylphenidate_ = 57, *p* < .001, and the modafinil‐only scores, *U* = 996.00, *N*_modafinil‐only_ = 86, *N*_control_ = 106, *p* < .001, were significantly lower compared with the control group.

### 3.3 Modafinil and methylphenidate

#### 3.3.1 Modafinil

The mean age of first use of modafinil stated by reported modafinil users was 28 years (SD = 9.24) with a range of 50 years (18 – 68). The only route of administration reported by this group was by oral ingestion.

There was almost an equal split between those who reported always taking the same dose (52%, *N* = 45) and those who reported not always taking the same dose (48%, *N* = 41). Frequency of use of modafinil can be found in Supplementary File S4. Full details of reported dosage levels can be found in Table 3.

**TABLE 3:** Dosage levels of reported modafinil use.

Dosage	*N* (%)^a^
Less than 50 mg	1 (1.16)
50 mg	13 (15.12)
100 mg	28 (32.56)
150 mg	7 (8.14)
200 mg	29 (33.72)
300 mg	3 (3.49)
400 mg	4 (4.65)
More than 500 mg	1 (1.16)

^a^ Percentage refers to group only.

Full details of the maximum and minimum reported dose of modafinil can be found in Table 4.

**TABLE 4:** Maximum and minimum reported dose of modafinil taken.

Dosage	Maximum *N* (%)^a^	Minimum *N* (%)^a^
**Less than 50** **mg**	–	15 (6.02)
**50** **mg**	1 (0.40)	31 (12.45)
**100** **mg**	14 (5.62)	25 (10.04)
**150** **mg**	7 (2.81)	3 (1.21)
**200** **mg**	36 (14.46)	11 (4.42)
**250** **mg**	–	–
**300** **mg**	8 (3.21)	1 (0.40)
**350** **mg**	–	–
**400** **mg**	16 (6.43)	–
**450** **mg**	2 (0.80)	–
**500** **mg**	2 (0.80)	–
**More than 500** **mg**	–	–

^a^ Percentages relate to group only.

### 3.4 Methylphenidate

The mean age of first use of methylphenidate was 21 years (SD = 6.15), with a range of 29 years (13 – 42). The majority of respondents reported that they did not always take the same dose (70%, *N* = 40). Dosage levels of reported methylphenidate use can be found in Table 5.

**TABLE 5:** Dosage levels of reported methylphenidate use.

Dosage	*N* (%)^a^
Less than 10 mg	3 (5.26)
10 mg	12 (21.05)
20 mg	14 (24.56)
30 mg	9 (15.79)
40 mg	6 (10.53)
50 mg	8 (14.04)
60 mg	2 (3.51)
70 mg	1 (1.75)
80 mg	2 (3.51)

^a^ Percentage refers to group only.

Full details of the maximum and minimum reported dose of methylphenidate can be found in Table 6.

**TABLE 6:** Maximum and minimum reported dose of methylphenidate use.

Dosage	Maximum *N* (%)^a^	Minimum *N* (%)^a^
Less than 10 mg	–	28.07 (16)
10 mg	1.75 (1)	42.11 (24)
20 mg	5.26 (3)	17.54 (10)
30 mg	22.81 (13)	8.77 (5)
40 mg	15.79 (9)	–
50 mg	17.54 (10)	3.51 (2)
60 mg	7.02 (4)	–
70 mg	‐	–
80 mg	5.26 (3)	–
More than 80 mg	8.77 (5)	–

^a^Percentages relate to group only.

The most commonly reported route of administration was reported to be swallowing a pill, although 14% reported snorting methylphenidate. The most commonly reported formulation of methylphenidate was extended release. Full details can be found in Table 7.

**TABLE 7:** Methylphenidate formulations.

Do you most often take immediate‐release or extended‐release formula?	*N* (%)^a^
Immediate release	19 (33.33)
Extended release	24 (42.11)
Don't know	14 (24.56)

^a^ Percentages relate to group only.

### 3.5 Self‐reports of cognition

#### 3.5.1 Adult ADHD Self‐Report Scale (ASRS)

On Part A, 34% (*N* = 29) of the modafinil‐only respondents, 49% (*N* = 28) of the methylphenidate respondents, and 11% (*N* = 12) of the control respondents scored at the level indicating symptoms highly consistent with ADHD.

A Kruskal–Wallis test was performed on the complete ASRS to test for the effect of group type (modafinil‐only, methylphenidate, and control). The differences between the mean ranks of 126.32 (the methylphenidate group), 119.84 (the modafinil‐only group), and 95.28 (the control group) were significant, *H*(_2_) = 9.58, *p* = .008. Post hoc Mann–Whitney *U* tests revealed that both the scores of the modafinil‐only group, *U* = 2707.50, *N*_modafinil‐only_ = 85, *N*_control_ = 82, *p* = .012, and the scores of the methylphenidate group, *U* = 1702.50, *N*_methylphenidate_ = 57, *N*_control_ = 82, *p* = .006, were significantly higher than the scores of the control group. There was no significant difference in scores between the modafinil‐only group and the methylphenidate group, *U* = 2269.00, N_modafinil‐only_ = 85, N_methylphenidate_ = 82 *p* = .521.

A Kruskal–Wallis test was performed on the complete ASRS inattentive scores to test for the effect of group type. The differences between the mean ranks of 130.32 (the methylphenidate group), 127.39 (the modafinil‐only group), and 86.37 (the control group) were significant, *H*(_2_) = 22.49, *p* < .001. Post hoc Mann–Whitney *U* tests revealed that both the modafinil‐only (*U* = 2211.00, *N*_modafinil‐only_ = 85, *N*_control_ = 83, *p* < .001), and the methylphenidate (*U* = 1472.00, *N*_methylphenidate_ = 57, *N*_control_ = 83, *p* < .001) groups identified self‐reported symptoms of inattentiveness compared with the control group's scores. However, the modafinil‐only group's scores were not significantly different to the methylphenidate group's scores, *U* = 2329.00, *N*_modafinil‐only_ = 85, *N*_methylphenidate_ = 57, *p* = .695.

A Kruskal–Wallis test was performed on the ASRS hyperactive/impulsive scores to test for the effect of group type. The differences between the mean ranks of the methylphenidate (116.82), modafinil‐only (107.05), and control (117.90) groups were not statistically significant, *H*(_2_) = 1.41, *p* = .494.

A Kruskal–Wallis test was performed on the separate inattentive and hyperactive ASRS scores to compare the scores of the three participant groups. The differences between the mean ranks of the methylphenidate group (130.32), the modafinil‐only group (127.39), and the control group (86.37) were significant, *H*(_2_) = 22.49, *p* < .001. Post hoc Mann–Whitney *U* tests revealed that both the inattentive scores of the modafinil‐only group (mean = 54.31, SD = 2.19), *U* = 2211.00, *N*_modafinil‐only_ = 85, *N*_control_ = 83, *p* < .001, and the inattentive scores of the methylphenidate group (mean = 4.53, SD = 2.39), *U* = 1472.00, *N*_methylphenidate_ = 57, *N*_control_ = 83, *p* < .001, were significantly higher compared with the control group's scores (mean = 2.99, SD = 1.55). There was no significant difference between groups for hyperactive/impulsive scores.

#### 3.5.2 Cognitive Failures Questionnaire

A Kruskal–Wallis test was performed on the CFQ scores to test for the effect of group type. The differences between the mean ranks of the methylphenidate group (72.41), the modafinil‐only group (82.76), and the control group (187.55) were significant, *H*(_2_) = 139.95, *p* < .001. Post hoc Mann–Whitney *U* tests revealed that the scores of both the modafinil‐only group scores (mean = 56.77, SD = 14.25) and the methylphenidate group scores (mean = 54.35, SD = 13.49) were significantly lower than the control group scores (mean = 85.28, SD = 13.06), *U* = 316.50, *N*_modafinil_ = 86, *N*_methylphenidate_ = 57, *N*_control_ = 106, *p* < .001.

#### 3.5.3 General Procrastination Scale

A Kruskal–Wallis test was performed on the GPS scores to compare the scores of the three participant groups. The differences between the mean ranks of the methylphenidate group (150.51), the modafinil‐only group (131.73), and the control group (105.83) were significant, *H*(_2_) = 15.42, *p* < .001. Post hoc Mann–Whitney *U* tests revealed that scores on the GPS were significantly higher for the both the modafinil‐only (mean = 61.06, SD = 13.14) and the methylphenidate groups (mean = 64.65, SD = 13.37) compared with the control group's scores (mean = 56.11, SD = 13.47), *U* = 1947.00, *N*_methylphenidate_ = 57, *N*_modafinil_ = 86, *N*_control_ = 106, *p* < .001.

### 3.6 ASRS, CFQ, and GPS

#### 3.6.1 Differences in age and gender

Due to differences in age and gender between the modafinil‐only, methylphenidate, and control groups, a series of 2 (gender) × 5 (education) × 3 (group type) unrelated ANOVAs were conducted on responses to each of the ASRS, CFQ and GPS. The only significant finding was for performance on the ASRS, with an interaction between gender and group type, *F*(3,199) = 4.34, MSE = 9.35, *p* = .018. Post hoc Mann–Whitney *U* tests revealed that the differences in scores by gender on the ASRS for all three groups were not significant. See Supplementary File S5 for details.

## 4 DISCUSSION

This study investigated the self‐perceived cognitive performance of self‐identified off‐prescription users of modafinil and methylphenidate among an international sample of individuals who were visitors of online forums. The results suggest that both the CED user groups reported significantly greater symptoms of inattention and procrastination and significantly lower cognitive failures than the control group. Scoring on both Part A of the ASRS and the whole questionnaire revealed that both the modafinil‐only group and the methylphenidate group reported symptoms highly consistent with ADHD but this pattern was not found for the control group. The modafinil‐only and methylphenidate group differences appeared to be mostly driven by items that measure inattention, not hyperactivity. It should be noted that these are self‐reported perceptions of respondents and, as such, may overreflect or underreflect prevalence rates of ADHD. However, this finding does suggest that reported modafinil and methylphenidate respondents feel that they have difficulties with attention that may be similar to those experienced by people with a diagnosis of ADHD. This finding is consistent with Peterkin et al. (2011) and Arria et al. (2011), while Francis et al. (2022) have also found that symptoms of inattention significantly predicted prescription stimulant misuse in college students with and without a diagnosis of ADHD. This result partially supports the hypothesis that self‐perceived modafinil and methylphenidate users will score significantly higher on the ASRS compared with controls and is consistent with the findings reported by Arria et al. (2011). Arria et al. (2011) argued that problems with inattention, rather than hyperactivity and impulsivity, are more likely to be associated with the nonprescription use of stimulants. They based this conclusion on finding a relationship between inattention and academic performance difficulties, but no relationship between hyperactivity/impulsivity and academic performance. Similarly, Rabiner et al. (2009) also reported an association between off‐prescription use of stimulants and symptoms of ADHD. They reported that students scoring high on attention difficulties were almost twice as likely to be nonmedical users of ADHD medications as students scoring lower on attention difficulties. Additionally, hyperactive/impulsive symptoms were not found to predict nonmedical ADHD use.

Analysis of the scores on the GPS revealed, as predicted, that both the modafinil‐only and methylphenidate groups scored significantly higher than the control group. This part of the hypothesis was therefore supported. Both Ferrari and Sanders (2006) and Niermann and Scheres (2014) reported associations between ADHD symptomology and self‐reported levels of procrastination, but neither research team looked specifically at CED‐using groups. Niermann and Scheres (2014) also reported that procrastination was only associated with ADHD symptoms of inattention and not hyperactivity and impulsivity. Further to this, in their event‐related potential study with low and high academic procrastinators, Michalowski et al. (2020) found that procrastinators have specific deficits in attention that can be observed at a neuronal level. It seems that the CED users who participated in the current study are self‐reporting cognitive performance that is in keeping with associations between inattention and procrastination but not hyperactivity and procrastination.

In contrast, however, both the modafinil‐only and methylphenidate groups scored significantly lower on the CFQ compared with the control group. If the CFQ is considered in relation to the factors identified by Wallace et al. (2002), which are memory, distractibility, blunders, and memory for names, the lower scores would suggest that off‐prescription users of modafinil and methylphenidate do not perceive themselves as having particular problems overall with these every day cognitive failures. In fact, it would suggest that these off‐prescription CED users perceive less problems with cognitive failures than other online forum users. One possible explanation is that those who may be self‐medicating might be doing so for problems with attention but not for the problems which the CFQ tests for, given that cognitive failures are considered to cover all conceivable everyday errors of memory, attention, and language (e.g., Broadbent et al., 1982; Unsworth et al., 2012). Self‐report questionnaires focusing on attention may be better attuned to probing these issues, such as the Everyday Life Attention Scale (Groen et al., 2018). The higher rates of alcohol use among the controls may also contribute to their higher scores on the CFQ, although previous research has linked increased cognitive failures among heavy drinkers more to experience of withdrawal from alcohol rather than its use per se (Carrigan & Barkus, 2016).

There were also some differences between CED users and nonusers in their reported use of alcohol, nicotine and cannabis. Previous studies have often reported higher rates of illicit drug use among CED users (McCabe et al., 2006), so questions on rates of illicit drug use were included to see if CED use is related to increased use of common recreational drugs. CED users were more likely to report being daily users of nicotine, higher lifetime cannabis use and their lower scores on the AUDIT suggest less frequent or less potentially problematic use of alcohol. The more frequent self‐perceived use of nicotine could be expected among CED users as nicotine can also act as a cognitive enhancer and it, therefore, might be used for this very same purpose by CED users (Heishman et al., 2010). The lower scores on the AUDIT, however, suggest that CED users may show more restraint in the use of a drug that is known to have cognitively impairing effects, particularly impacting on working memory, encoding and prospective memory (memory for delayed intentions; Winograd, 1988), abilities CED users may be trying to improve (Van Skike et al., 2019). Cannabis has well documented acute effects on cognition, including as measured by the ASRS (Petker et al., 2020); therefore, current and frequent use could, in theory, lead to higher self‐perceived inattention and procrastination. The CED users and controls only differed in cannabis use over their lifetime.

While giving important insights into the self‐rated cognitive performance the current study does, however, have a limitation. Self‐reports of cognitive and behavioral performance are subjective perceptions that have been demonstrated to differ quite substantially from performance as measured by objective tests (Ilieva & Farah, 2019) and this must be taken into consideration when interpreting the results. That said, this may be due to the fact that they measure performance at different levels (Stanovich, 2009). Stanovich (2009) argued that performance‐based objective tests under laboratory conditions measure optimal performance whereas self‐report rating measures assess typical performance in everyday life. The data reported here, do, however, suggest that perceived poorer cognitive performance may be related to CED use but objective, laboratory‐based tests would be needed to be certain that CED users genuinely do experience poorer attentional control and higher levels of procrastination.

## 5 CONCLUSION

These findings suggest that some reported off‐prescription users of modafinil and methylphenidate (at least those who frequent online forums) are self‐prescribing for perceived problems with inattention and procrastination and that these drugs are perceived as improving these problems. This finding has implications, which are important, not only in informing policy, but also in highlighting the possible existence of a population of CED users who may struggle with undiagnosed ADHD. Able et al. (2007) and Okumura et al. (2021) have raised the point that individuals with undiagnosed ADHD manifest functional and psychosocial impairments which create a significant burden in their lives. Additionally, as argued by Scope et al. (2010), inattention may exist along a continuum and, as such, there may be CED‐using individuals who suffer with subclinical levels of inattention and, by extension, procrastination. Further research is needed to determine whether these subjectively perceived problems are reflected via objective measures.

## CONFLICT OF INTEREST STATEMENT

There are no conflicts of interest to declare.

### PATIENT CONSENT STATEMENT FOR WORKING WITH HUMAN SUBJECTS

As this was an online survey, a consent form was presented requiring all statements indicating consent to be selected before the survey commenced.

### PEER REVIEW

The peer review history for this article is available at https://publons.com/publon/10.1002/brb3.3403.

## Supporting information

Supporting Information

Supporting Information

Supporting Information

Supporting Information

Supporting Information

## Data Availability

The data from this study is available in the S10 file attached.
