# Supplementary material for: Perceived cognitive performance in off‐prescription users of modafinil and methylphenidate: an online survey
Source: Brain Behav. 2024 Feb 4;14(2):e3403. doi: 10.1002/brb3.3403 (PMC10839162; doi:10.1002/brb3.3403)
Supplement: Supplementary file 1 — Supporting Information [file BRB3-14-e3403-s002.docx]

**List of subReddit forum sites**

**Reddit forum sites where the survey was advertised**

r/Nootropics

r/afinil

r/Stims

r/AskDrugNerds

r/StackAdvice

r/darknet

r/Drugs

r/UKUniversityStudents

r/UniUK

r/lifelonglearning

r/Scholar

r/explainlikeimfive

r/showerthoughts

r/samplesize
