# Supplementary material for: Perceived cognitive performance in off‐prescription users of modafinil and methylphenidate: an online survey
Source: Brain Behav. 2024 Feb 4;14(2):e3403. doi: 10.1002/brb3.3403 (PMC10839162; doi:10.1002/brb3.3403)
Supplement: Supplementary file 2 — Supporting Information [file BRB3-14-e3403-s005.docx]

**S13 File**

The principal component analysis (using a varimax rotation) resulted in six factors with all items loading on one factor and only two items loading on two factors. Additionally, Wallace et al.’s (2002) Distractibility factor consisted of nine items and the current factor analysis identified one factor comprising of seven of the same items, including Item 1 (the unanswered question). As this factor consists of most of the same items, the mean score for all other items was calculated to provide an accurate estimate of the missing response.

## **Factor analysis and scree plot of CFQ control group responses**

**Factor loading of control group CFQ responses**

| **Item number** | **Factor 1** | **Factor 2** | **Factor 3** | **Factor 4** | **Factor 5** | **Factor 6** |
| --- | --- | --- | --- | --- | --- | --- |
| CFQ.16 | .696 |  |  |  |  |  |
| CFQ.17 | .668 |  |  |  |  |  |
| CFQ.6 | .544 |  |  |  |  |  |
| CFQ.2 | .509 | .475 |  |  |  |  |
| CFQ.3 | .498 |  |  |  |  |  |
| CFQ.11 | .469 |  |  |  |  |  |
| CFQ.23 |  |  |  |  |  |  |
| CFQ.9 |  |  |  |  |  |  |
| CFQ.1 |  | .869 |  |  |  |  |
| CFQ.21 |  | .615 |  |  |  |  |
| CFQ.19 |  | .558 |  |  |  |  |
| CFQ.22 |  | .515 |  |  |  |  |
| CFQ.25 |  | .490 |  |  |  |  |
| CFQ.15 |  | .416 |  |  |  |  |
| CFQ.14 |  |  | .575 |  |  |  |
| CFQ.13 | .512 |  | .543 |  |  |  |
| CFQ.10 |  |  | .528 |  |  |  |
| CFQ.8 |  |  | .411 |  |  |  |
| CFQ.18 |  |  |  | .613 |  |  |
| CFQ.4 |  |  |  | .561 |  |  |
| CFQ.24 |  |  |  | .447 |  |  |
| CFQ.12 |  |  |  |  |  |  |
| CFQ.7 |  |  |  |  | .871 |  |
| CFQ.20 |  |  |  |  | .588 |  |
| CFQ.5 |  |  |  |  |  | .606 |


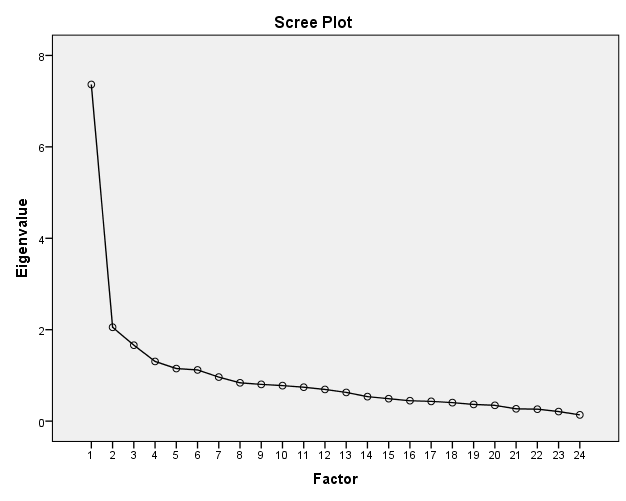


**Figure X. Scree plot of eigenvalues for CFQ factors**
