# Supplementary material for: Perceived cognitive performance in off‐prescription users of modafinil and methylphenidate: an online survey
Source: Brain Behav. 2024 Feb 4;14(2):e3403. doi: 10.1002/brb3.3403 (PMC10839162; doi:10.1002/brb3.3403)
Supplement: Supplementary file 3 — Supporting Information [file BRB3-14-e3403-s001.docx]

**Cannabis**

For the purpose of analysing the data collected on cannabis use, a clearer understanding of the frequency of use of cannabis was required. Therefore, responses to the question ‘in the past six months how regularly have you taken cannabis’ were condensed; ‘everyday/ almost everyday’, ‘three to four times per week’ and ‘once per week’ were grouped into the variable ‘once or more per week’. The responses ‘once or twice per month’ and ‘up to three times in total’ were grouped into the variable ‘less than once per week’ and the response ‘none’ was renamed ‘none in the past six month’ for clarity.

The methylphenidate group reported higher lifetime use of cannabis (methylphenidate group = 98%, N = 56; modafinil-only group = 81%, N = 70; control group = 60%, N = 64). The methylphenidate group also reported more frequent, current use of cannabis (for everyday/almost everyday use, methylphenidate group = 21%, N = 12; modafinil-only group = 9%, N = 8; control group = 8%, N = 9). The least frequent use of cannabis was reported by the modafinil-only group (for no use in the past six months, modafinil-only group N = 31, 36%; methylphenidate group N = 10, 18%, control group N = 21, 20%).

Chi-square analyses were conducted on all three group types for lifetime use of cannabis. To reduce the chance of Type 1 errors occurring, Bonferroni corrections were used, resulting in a critical value of .017. The Chi-square analysis revealed significant associations between lifetime use of cannabis and group membership (methylphenidate vs. control: χ^2^(1, N = 163) = 27.37, *p* < .001; modafinil vs. control: χ^2^(1, N = 192) = 9.95, *p* = .002; methylphenidate vs. modafinil: χ^2^(1, N = 143) = 9.29, *p* = .002). Lifetime use of cannabis was more likely in the methylphenidate and modafinil-only groups and less likely in the control group. In addition, the two CED groups differed from each other, with lifetime use of cannabis more likely in the methylphenidate group as compared to the modafinil group.

Chi-square analyses were also conducted to test for the association between group type and frequency use of cannabis. Bonferroni corrections were applied, resulting in a critical value of .001. The Chi-square analyses revealed that none of the comparisons were significant (all *p*.’s > .017).

**Frequency of use of cannabis within the past six months**

**Chi-square analysis for the modafinil-only and control groups**

The Chi-square analysis found that the association for the frequency of use of cannabis between the modafinil-only group and the control group was not significant, χ^2^(5, N = 134) = 2.24, *p* = .816.

**Chi-square analysis for the methylphenidate and control groups**

The Chi-square analysis found that the association for the frequency of use of cannabis between the methylphenidate group and the control group was not significant, χ^2^(5, N = 120) = 5.57, *p* = .350.

**Chi-square analysis for the methylphenidate and modafinil-only groups**

The Chi-square analysis found that the association for the frequency of use of cannabis between the methylphenidate group and the modafinil-only group was not significant, χ^2^(5, N = 126) = 13.82, *p* = .017.

Details of the frequency of use of cannabis within the last six months can be found in Table 1.

**Table 1. Frequency of use of cannabis within the last six months**

| **In the past six months, how regularly have you taken cannabis?** | **Modafinil-only**  **N (%)*** | **Methylphenidate**  **N (%)*** | **Control N (%)*** |
| --- | --- | --- | --- |
| **Every day/ almost every day** | 8 (9.30) | 12 (21.05) | 9 (8.49) |
| **3-4 times per week** | 5 (5.81) | 11 (19.30) | 7 (6.60) |
| **Once per week** | 8 (9.30) | 4 (7.02) | 7 (6.60) |
| **1-2 times per month** | 6 (6.98) | 7 (12.28) | 7 (6.60) |
| **Up to 3 times in total** | 12 (13.95) | 12 (21.05) | 13 (12.26) |
| **None** | 31 (36.05) | 10 (17.54) | 21 (19.81) |

*** Percentage refers to group only**

**Nicotine**

Due to a technical error in Qualtrics, responses were only collected in the ‘everyday/almost everyday’ option for the frequency of use question and the ‘today’ options for the question relating to the length of time since nicotine was last used. The modafinil-only group reported the highest level of everyday use of nicotine (53%, N = 46), followed closely by the methylphenidate group (42%, N = 24) compared with controls (21%, N = 22).

A Kruskal-Wallis test was performed on the data for everyday use of nicotine to test for the effect of group type. To avoid Type 1 errors, the Bonferroni correction was again used, resulting in a critical value of .025. The differences between the mean ranks of 140.95 (methylphenidate), 158.60 (modafinil-only) and 89.17 (control) were significant, *H(2)* = 63.72, *p* < .001.

*Post hoc* Mann-Whitney U tests revealed that the modafinil-only group, *U* = 2016.00, N_modafinil-only_ = 86, N_control_ = 106, *p* < .001, and the methylphenidate group, *U* = 1764.50, N_methylphenidate_ = 57, N_control_ = 106, *p* < .001, reported significantly higher levels of everyday use of nicotine compared with controls.

**Everyday use of nicotine**

A Kruskal-Wallis test was performed on the data for everyday use of nicotine to test for the effect of group type. To avoid Type 1 errors, the Bonferroni correction was again used, resulting in a critical value of .025. The differences between the mean ranks of 140.95 (methylphenidate), 158.60 (modafinil-only) and 89.17 (control) were significant, *H(2)* = 63.72, *p* < .001.

*Post hoc* Mann-Whitney U tests revealed that the modafinil-only group, *U* = 2016.00, N_modafinil-only_ = 86, N_control_ = 106, *p* < .001, and the methylphenidate group, *U* = 1764.50, N_methylphenidate_ = 57, N_control_ = 106, *p* < .001, reported significantly higher levels of everyday use of nicotine compared with controls.

Of the methylphenidate group, 61.4% reported everyday use of nicotine, of the modafinil-only group, 75.6% reported everyday use of nicotine, whereas, of the control group, just 19.8% reported everyday use of nicotine.
