# Supplementary material for: Perceived cognitive performance in off‐prescription users of modafinil and methylphenidate: an online survey
Source: Brain Behav. 2024 Feb 4;14(2):e3403. doi: 10.1002/brb3.3403 (PMC10839162; doi:10.1002/brb3.3403)
Supplement: Supplementary file 4 — Supporting Information [file BRB3-14-e3403-s004.docx]

**Frequency of use of modafinil**

**“*In the past six months, how regularly have you taken Modafinil?*”**

| **Frequency** | **N (%)** |
| --- | --- |
| Every day/almost every day | 21 (16.9) |
| 3-4 times per week | 41 (33.1) |
| Once per week | 20 (16.1) |
| 1-2 times per month | 15 (12.1) |
| Up to 3 times in total | 16 (12.9) |
| None | 11 (8.9) |

(N = 124)
