# Supplementary material for: Perceived cognitive performance in off‐prescription users of modafinil and methylphenidate: an online survey
Source: Brain Behav. 2024 Feb 4;14(2):e3403. doi: 10.1002/brb3.3403 (PMC10839162; doi:10.1002/brb3.3403)
Supplement: Supplementary file 5 — Supporting Information [file BRB3-14-e3403-s003.docx]

**ASRS, CFQ and GPS**

**Differences in age and gender**

Due to differences in age and gender between the modafinil-only, methylphenidate and control groups a series of 2 (gender) x 5 (education) x 3 (group type) unrelated ANOVAs were conducted on performance on the ASRS, CFQ and GPS. Bonferroni corrections resulted in an alpha level of .007.

**ASRS Total**

The means and SDs for gender by group type for performance on the ASRS are found in Table 1 and the means and SDs for education by group type for performance on the ASRS are found in Table 2.

**The main effect of drug user type was significant**, *F*(_2,199_) = 6.12, MSE = 9.35, *p* = .003. *Post hoc* Mann-Witney *U* tests indicated that there was a significant difference between the **methylphenidate group and control group** for the performance on the ASRS, *U* = 1372.00, N_methylphenidate_ = 57, N_control_ = 83, *p* < .001 and there was a significant difference between the **modafinil-only group and control group** for performance on the ASRS, *U* = 2259.50, N_modafiil-only_ = 85, N_control_ = 83, *p* < .001. However, the difference between the modafinil-only and methylphenidate groups was not significant, *p* = .367.

The main effects for gender and education were not significant, all *p*s > .05. The three-way interaction between gender, group type and education was not significant, *F*(6,199) = 1.92, MSE = 9.35, *p* = .079. The interaction between gender and group type was also not significant, *F*(3,199) = 4.34, MSE = 9.35, *p* = .018.

However, the interaction between **gender and education** was significant, *F*(_3,199_) = 4.34, MSE = 9.35, *p* = .005.

**Table 1: Means and SDs for gender**

|  | **MPH** | | **MOD** | | **Control** | |
| --- | --- | --- | --- | --- | --- | --- |
|  | **N** | **M (SD)** | **N** | **M (SD)** | **N** | **M (SD)** |
| **Male** | 44 | 50.75 (10.03) | 68 | 48.47 (9.50) | 45 | 43.87 (7.30) |
| **Female** | 12 | 51.83 (12.88) | 15 | 53.40 (10.82) | 37 | 43.51 (8.96 |

**Table 2: Means and SDs for education**

|  | **MPH** | | **MOD** | | **Control** | |
| --- | --- | --- | --- | --- | --- | --- |
|  | **N** | **M (SD)** | **N** | **M (SD)** | **N** | **M (SD)** |
| **No formal education** | 1 | 59.00 (0) | 1 | 46.00 (0) | 0 | 0 |
| **Educated to age 16** | 1 | 60.00 (0) | 0 | 0 | 2 | 39.50 (3.54) |
| **Educated to age 18** | 32 | 51.72 (11.99) | 22 | 51.86 (10.84) | 37 | 43.68 (7.64) |
| **University Degree (BA/BSc)** | 16 | 49.31 (8.72) | 45 | 47.87 (8.83) | 25 | 44.08 (9.14) |
| **Post-graduate Degree (MA/ MSc/PhD** | 7 | 49.71 (7.93) | 17 | 50.18 (11.30) | 19 | 43.26 (7.99) |

**ASRS Inattentive and Hyperactive/Impulsive**

There were no significant main effects or interactions for the ASRS subscales of inattention and hyperactivity/impulsivity.

**CFQ**

The means and SDs for gender by group type for performance on the CFQ are found in Table 1 and the means and SDs for education by group type for performance on the CFQ are found in Table 2.

**Table 1: Means and SDs for gender**

|  | **MPH** | | **MOD** | | **Control** | |
| --- | --- | --- | --- | --- | --- | --- |
|  | **N** | **M (SD)** | **N** | **M (SD)** | **N** | **M (SD)** |
| **Male** | 44 | 56.23 (12.68) | 68 | 57.31 (14.13) | 58 | 84.43 (1.69) |
| **Female** | 12 | 48.03 (15.38) | 16 | 55.81 (15.48) | 46 | 86.11 (2.00) |

**Table 2: Means and SDs for education**

|  | **MPH** | | **MOD** | | **Control** | |
| --- | --- | --- | --- | --- | --- | --- |
|  | **N** | **M (SD)** | **N** | **M (SD)** | **N** | **M (SD)** |
| **No formal education** | 1 | 58.00 | 1 | 57.00 | 0 | 0 |
| **Educated to age 16** | 1 | 33.00 | 0 | 0 | 3 | 90.67 (5.24) |
| **Educated to age 18** | 32 | 54.44 (13.81) | 23 | 53.09 (13.81) | 45 | 87.31 (12.50) |
| **University Degree (BA/BSc)** | 16 | 52.38 (10.84) | 45 | 57.56 (13.92) | 35 | 82.29 (13.96) |
| **Post-graduate Degree (MA/ MSc/PhD** | 7 | 61.00 (16.69) | 17 | 59.65 (15.90) | 23 | 85.17 (12.92) |

**The main effect of drug user type was significant**, *F*(_2,224_) = 45.34, MSE = 186.37, *p* < .001. *Post hoc* Mann-Witney *U* tests indicated that there was a significant difference between the **methylphenidate group and control group** for the performance on the CFQ, *U* = 316.50, N_methylphenidate_ = 57, N_control_ = 106, *p* < .001 and there was a significant difference between the **modafinil-only group and control group** for performance on the CFQ, *U* = 632.50, N_modafiil-only_ = 86, N_control_ = 106, *p* < .001. However, the difference between the modafinil-only and methylphenidate groups was not significant, *p* = .227.

There were no other significant main effects or interactions, all *p*s > .05.

**GPS**

The means and SDs for gender by group type for performance on the GPS are found in Table 1 and the means and SDs for education by group type for performance on the GPS are found in Table 2.

The **main effect of education was significant**, *F*(_2,224_) = 4.02, MSE = 170.41, *p* = .004

There were no other significant main effects or interactions, all *p*s > .05.

There were no other significant main effects or interactions, all *p*s > .05.

**Table 1: Means and SDs for gender**

|  | **MPH** | | **MOD** | | **Control** | |
| --- | --- | --- | --- | --- | --- | --- |
|  | **N** | **M (SD)** | **N** | **M (SD)** | **N** | **M (SD)** |
| **Male** | 44 |  | 68 |  | 45 |  |
| **Female** | 12 |  | 15 |  | 37 |  |

**Table 2: Means and SDs for education**

|  | **MPH** | | **MOD** | | **Control** | |
| --- | --- | --- | --- | --- | --- | --- |
|  | **N** | **M (SD)** | **N** | **M (SD)** | **N** | **M (SD)** |
| **No formal education** | 1 | 83.00 | 1 | 74.00 | 0 | 0 |
| **Educated to age 16** | 1 | 75.00 | 0 | 0 | 2 | 54.00 (21.21) |
| **Educated to age 18** | 32 | 68.72 (12.77) | 23 | 62.09 (14.38) | 37 | 57.11 (12.40) |
| **University Degree (BA/BSc)** | 16 | 69.19 (13.90) | 45 | 59.78 (12.07) | 25 | 60.44 (14.60) |
| **Post-graduate Degree (MA/ MSc/PhD** | 7 | 52.57 (13.28) | 17 | 62.29 (14.62 ) | 19 | 58.11 (14.97) |

55 85 81
